# The association between leisure activity patterns and the prevalence of mild cognitive impairment in community-dwelling older adults

**DOI:** 10.3389/fpsyg.2022.1080566

**Published:** 2023-01-13

**Authors:** Yuanyuan Zhang, Xinxin Yang, Linlin Guo, Xinyi Xu, Bingyu Chen, Xiaowei Ma, Yan Li

**Affiliations:** ^1^College of Nursing, Hebei Medical University, Shijiazhuang, Hebei, China; ^2^Neuroscience Research Center, Hebei Medical University, Shijiazhuang, Hebei, China; ^3^Hebei Key Laboratory of Neurodegenerative Disease Mechanism, Shijiazhuang, Hebei, China; ^4^Department of Anatomy, Hebei Medical University, Shijiazhuang, Hebei, China; ^5^Department of Neurology, Hebei Medical University First Affiliated Hospital, Shijiazhuang, Hebei, China

**Keywords:** leisure activities, mild cognitive impairment, latent class analysis, older adult, nursing

## Abstract

**Objectives:**

The study aimed to identify leisure activity patterns among older adults and explore the differences in the prevalence of mild cognitive impairment (MCI) of these patterns.

**Methods:**

A total of 352 older adults aged ≥ 65 years in Shijiazhuang were enrolled in this study from June to September 2021. Their leisure activities and cognition were evaluated. Latent class analysis and logistic regression models were used for analysis. The manuscript was organized according to the STROBE guidelines.

**Results:**

We identified three latent classes of leisure activities: multidomain activities (MDA) class (55%), self-improvement activities (SIA) class (21%), and family-centered activities (FCA) class (24%). Three latent classes significantly differed in general characteristics (gender, education, and body mass index) and the prevalence of MCI. The prevalence of MCI was 3.694 times higher in older adults in the FCA class than in older adults in the MDA class (OR = 3.694, *P* < 0.001) and 2.573 times higher in older adults in the SIA class (OR = 2.573, *P* = 0.036).

**Conclusion:**

Patterns of low participation in intellectual activities were associated with MCI. Identifying the heterogeneity in leisure activity engagement in later life was critical for tailoring interventions and designing programs that can improve the cognitive function of older adults.

## 1. Introduction

Mild cognitive impairment (MCI) describes an intermediate state between normal aging and dementia (Xue et al., 2018). MCI is a deficit in one or more cognitive domains, with relative preservation of functional independence without dementia (Petersen et al., 1999). The global prevalence of MCI in older adults over 60 years old is 5.0 ~ 36.7% (Dementia Cognitive Disorders Group, 2022). Older adults with MCI are more likely to progress to dementia than their cognitively normal counterparts (Shi-xiang and Yan-jie, 2017). 60.5% of MCI patients will develop dementia within 5 years after the initial diagnosis of MCI (Manly et al., 2008). Compared with irreversible damage of dementia, early intervention in patients with MCI can delay or prevent dementia development and even possibly reverse it to a normal cognitive level (Jin et al., 2017; Pandya et al., 2017). Therefore, MCI may be an important stage for the early detection and intervention of dementia.

There are numerous risk factors for MCI progression, including inheritance, demographic, metabolism, lifestyle, and biological factors. However, the main modifiable risk factor is a leisure activity in lifestyle (Livingston et al., 2020; Chowdhary et al., 2021). Leisure activities are defined as activities in which individuals participate for enjoyment, these activities are independent of work, and mainly include physical, social, and intellectual activities (Verghese et al., 2006; Yang et al., 2015). For retirees, leisure activities are an important part of their lifestyle. Leisure activity engagement may become a crucial part of the adjustment for retirees to maintain cognitive function postretirement (Lee et al., 2018).

Numerous previous studies have found that leisure activity was associated with cognition (Li et al., 2021; Sharifian et al., 2021). Some studies have focused on the association between single leisure activity and cognition and mainly explored the improvement effect of a single activity on cognition. They have found that single activities, such as gardening (Park et al., 2020), square dancing (Zhao et al., 2021), and Tai Chi (Li et al., 2022) can improve cognition in older adults. Additionally, some studies have focused on a certain type of activity, such as physical activity, social activity, and intellectual activity, and mainly analyzed the association between leisure activity and cognition from different dimensions. They have provided evidence that activity, regardless of its type, generally was positively associated with cognition in older adults (Kim et al., 2017; Ingold et al., 2020; Kurita et al., 2020). These above-mentioned studies provide a research basis for exploring the association between leisure activities and cognition and also make important contributions. However, considering that most older adults engage in multiple activities simultaneously (Morrow-Howell et al., 2014), using one activity or a set of certain leisure activity types as a measure may not offer a full picture of an individual's activity engagement patterns. Therefore, it is more realistic to analyze the leisure activity patterns of older adults from the overall activity participation characteristics and then explore the association between different patterns and cognition.

Latent class analysis (LCA) (Qiu, 2008) offers considerable promise in identifying activity patterns. LCA can identify smaller homogeneous subgroups, i.e., classes, based on all types of different leisure activities pursued by older adults. So that we can tailor matching interventions to the unique characteristics and needs of older adults. Fewer studies have investigated activity patterns based on the rationale that older adults can and do participate in multiple activities simultaneously (Amano et al., 2018; Chen et al., 2019; Zhang et al., 2020; Katayama et al., 2021). Moreover, these studies explored the association between each pattern and cognitive levels and did not focus on the MCI stage, a critical status of cognitive decline and cognitive intervention. Furthermore, due to the influence of different cultures, customs, and religious beliefs in different regions, there are certain differences in the types of leisure activities that older adults engage in, resulting in different leisure activity patterns discussed in each study (Amano et al., 2018; Chen et al., 2019; Zhang et al., 2020; Katayama et al., 2021). Therefore, further studies on the association between leisure activity patterns and MCI risk in the Chinese cultural context are warranted. This has important implications for promoting global healthy aging.

By using latent class modeling, the current study aimed to identify leisure activity patterns of older adults in China, explore the influencing factors of different patterns, and further estimate the association between these patterns and the prevalence of MCI. The results of this study would allow health professionals to customize specific interventions for older adults with different characteristics and needs in the future.

## 2. Population and methods

A cross-sectional design was used. According to the 10EPV (Events per variable) rule (Peduzzi et al., 1996), each independent variable should have at least ten samples. In our research, there were 30 variables (23 related to items of leisure activities, four related to demographic information, and three related to physical condition). Therefore, the minimum sample size in this study is at least 300. This study was approved by the Ethics Committee of Hebei Medical University (ethical approval number 2021080), and written informed consent was obtained from each subject before the experiment. This study was reported according to the STROBE Statement: Guidelines for Reporting Observational Studies (Cuschieri, 2019).

### 2.1. Study population

#### 2.1.1. Recruitment and sampling method

Community health centers are grass-roots health institutions set up by the government in communities. They serve communities, families, and residents and focus on providing public health and basic medical services for women, children, the elderly, chronic patients, the disabled, and poor residents. According to national policy, permanent residents aged ≥ 65 years can have free annual medical examinations in the community. In this study, a community health service center was randomly selected from each of the four main urban areas in Shijiazhuang, Hebei Province, China, by cluster sampling. The head of each community center was contacted by telephone. After obtaining consent, a residential area under the jurisdiction was randomly selected from each of the four community health service centers. The community staff informed older adults to come to the community for free physical examination from June to September 2021. After reading a consent form outlining the purpose of the survey, its confidentiality, and the voluntary nature of participation, a field survey was conducted among the elderly volunteers. A total of more than 600 older adults aged 65 years or above who came to the community center for physical examination were screened. After the exclusion of cerebrovascular disease, cancer, liver disease, renal insufficiency, thyroid disease or other systemic diseases, depression symptoms, or hearing loss, 458 subjects were selected. Four hundred five older adults met the inclusion criteria, so a total of 405 questionnaires were issued, and 352 valid questionnaires were collected, with a recovery rate of 86.91%.

#### 2.1.2. Inclusion criteria

The inclusion criteria were as follows: (1) older adults aged ≥ 65 years; (2) older adults performing normal activities of daily living [the Activity of Daily Living (ADL) scale ≤ 26 (Sun et al., 2019)]; (3) older adults without dementia [the Mini-Mental State Examination (MMSE): illiterate > 17, primary school education > 20, and junior high school and above > 24 (Zhang, 1993)]; (4) older adults who voluntarily agreed to participate in the screening.

#### 2.1.3. Exclusion criteria

After the informed consent of older adults, we retrieved their health files in the community and extracted their past medical history. We needed to exclude any secondary cognitive impairment (not age-related) due to a disease. The exclusion criteria were as follows: (1) older adults with cerebrovascular disease; (2) older adults with liver disease, renal insufficiency, thyroid disease, and other systemic diseases; (3) older adults with cancer; (4) older adults with depressive symptoms; (5) those who cannot communicate well due to hearing loss.

### 2.2. Collected data

#### 2.2.1. General characteristic

The following self-reported demographic variables of the study subjects were assessed in this study: age, gender, education, and previous occupation. At the same time, after obtaining the participants' informed consent, we retrieved their health files in the community and extracted their history of hypertension and diabetes, as well as the values of height and weight. Body mass index (BMI) was calculated directly from weight and height records [weight (kg)/height (m^2^)]. Age, education, and BMI were reported as continuous variables. Gender was reported as a binary variable: male/female. The previous occupation was also reported as a binary variable: mental labor/manual labor. The history (yes/no) of hypertension and diabetes were included as binary variables in all models.

#### 2.2.2. Leisure activities

Leisure activity participation was evaluated by the questionnaire on the leisure activities of older Chinese adults (Yuanyuan et al., 2022). The questionnaire has relatively rich items, which are in line with the characteristics of the activities of the elderly in China. The questionnaire consists of 21 items, including walking, Kung fu (Tai Chi/martial art), square dancing, shopping, traveling, going to college for the aged, handwriting, painting, playing mahjong, and keeping pets. The questionnaire has good reliability and validity. The content validity index for each questionnaire item (I–CVI) was between 0.85 and 1, and that for the total questionnaire (S–CVI) was 0.973. Cronbach's α coefficient was 0.625 for the physical dimension, 0.605 for the social dimension, 0.625 for the cognitive dimension, and 0.856 for the total questionnaire. Many previous studies have not included housework, but the survey shows that cooking, housework, and babysitting represent the current status of activities of some older Chinese adults (Ning, 2020). Therefore, the exclusion of family-related activities may be an omission (Hong et al., 2020). To more comprehensively and accurately identify the activity patterns of the elderly, this study also included these three items in the assessment content. The internal consistency of this scale (Cronbach's a) was 0.701 in the present study.

Individuals indicated the frequency of participation in each activity on a three-point Likert scale (Zhu et al., 2017) (0 = never or less than once a month; 1 = once a month or more but less than once a week; 2 = once a week or more but less than once a day; 3 = almost every day). The frequency of participation in traveling was scored as “0 = never or less than once a year, 1 = once a year, 2 = 2–3 times a year, and 3 = more than 3 times a year.” Finally, for data analysis, each item was converted into a binary variable, with “0” representing no participation and “123” representing participation.

#### 2.2.3. Assessment of activities of daily living

Activities of daily living were examined by the ADL Scale. The scale mainly assesses the physical function and the ability to use tools in older adults. The scale includes 20 activities of daily living, including eating, dressing and undressing, bathing, toileting, feeding self, and grocery shopping. To ensure the accuracy of the information, the well-trained researchers asked the subject's primary caregiver as a good informant to rate the daily life ability of older adults. The 20 activities were rated as follows: 1: can do, 2: some difficulty but can do, 3: need some help, and 4: cannot do on their own. The lower the score, the better the ability of daily living. A total ADL score ≤ 26 is considered normal, whereas a score >26 indicates dysfunction (Sun et al., 2019).

#### 2.2.4. Neuropsychological assessment and diagnostic criteria

Global cognition was assessed by the MMSE and Montreal Cognitive Assessment (MoCA) (Beijing version) (Lu et al., 2011). In this study, the MMSE scale was used to exclude people with dementia. MoCA was designed as the main observation index for MCI screening (Roalf et al., 2013). Moreover, five different cognitive domains of older adults were also assessed. The auditory Verbal Learning Test (AVLT) was used to evaluate memory function, including immediate recall, long delay free recall, long delay cued recall, and long delay recognition function. The Boston Naming Test (BNT) was applied to evaluate the language function. The visuospatial function was evaluated by the Clock Drawing Test (CDT). Attention was assessed with the Digit Span Test (DST), which includes Digit Span Forward (DSF) and Digit Span Backward (DSB). The executive function was assessed with the Trail Making Test (TMT), which includes TMT-A and TMT-B parts. The time difference between TMT-B and TMT-A (TMT B-A) was used to assess executive function (Hirota et al., 2010). Additionally, the ADL (Sun et al., 2019) and Geriatric Depression Scale (GDS) (GDS > 10) (Yesavage et al., 1982) were used to exclude unqualified subjects. All the above-mentioned tests were performed by well-trained researchers in this study.

This diagnosis of MCI was made by an experienced neurologist according to the Peterson diagnostic criteria (Knopman and Petersen, 2014). The subjects met the following criteria: (1) the patient complained of memory impairment, which was confirmed by an insider; (2) objective evidence of impairment in one or more cognitive domains (MoCA: illiterate ≤ 13, primary school ≤ 19, and junior high school and above ≤ 24) (Lu et al., 2011); (3) normal or slightly impaired activities of daily living (ADL ≤ 26) (Sun et al., 2019); (4) non-dementia (MMSE: illiterate > 17, primary school education > 20, and junior high school and above > 24) (Zhang, 1993).

### 2.3. Statistical analysis

LCA was performed by MPlus version 8.3 software. Two types of model fitting test indicators were applied to determine the optimal number of latent classes: (1) information evaluation indicators: Akaike Information Criteria (AIC), Bayesian Information Criteria (BIC), Adjusted Bayesian Information Criteria (aBIC), and Entropy; (2) likelihood ratio test indicators: Lo-Mendell-Rubin Likelihood Ratio Test (LMRT), and Bootstrapped Likelihood Ratio Test (BLRT). The smaller the values of AIC, BIC, and aBIC, the better the model fitting effect. Entropy represents the classification accuracy, and the value is between 0 and 1. The closer entropy is to 1, the higher the classification accuracy. When the value exceeds 0.8, the classification accuracy often exceeds 90% (Mengcheng and Xiangyang, 2018). If the *p*-values of LMRT and BLRT reach a significant level, it indicates that the model with K classes is better than the model with K-1 classes. Due to the extreme situation of data distribution, practice handwriting and painting were combined into one activity.

SPSS 26.0 was used for further analysis. Continuous variables with normal distribution were expressed as mean ± standard deviation (SD), and those with skewed distribution were expressed as median (interquartile range). Categorical variables were expressed as frequencies and percentages. The χ^2^ test was used for the comparison of categorical variables between the groups, and the analysis of variance or non-parametric test was used for the comparison of continuous variables. Then, the multivariate Logistic regression model was used, and the latent classes were used as the dependent variable to explore the differences in characteristics of older adults with different leisure activity patterns. Finally, a binary logistic regression model was used to explore the association between the prevalence of MCI and different leisure activity patterns. Statistical tests used a significance level of α = 0.05.

## 3. Results

### 3.1. Sample characteristics

A total of 352 older adults (201 males/151 females) aged 65–88 years were included. The prevalence of MCI in this study was 18.75% (*n* = 66). The subjects had a mean of 8.54 (SD = 3.96) years of formal education ranging from 0 to 20 years. The mean BMI of all participants was 25.31 (SD = 3.17). A total of 183 (52.0%) older adults were previously engaged in mental work, and 169 (48.0%) older adults were engaged in manual work. People with hypertension accounted for 45.5%, and people with diabetes accounted for 25.3%.

### 3.2. Latent classes of leisure activity engagement

This study constructed 1–5 latent class models based on 23 common leisure activities. The results of LCA model fitting indicators were shown in Table 1. With the increase in the number of model classes, the value of AIC and aBIC gradually decreased, and the aBIC value of M4 was the smallest. However, the *p*-value of LMRT in M4 could not reach a significant level, which suggested that the model-fitting effect of M4 was not as good as that of M3. Moreover, the entropy value of M4 was smaller than M3, which indicated that the classification accuracy of M4 was not as good as that of M3. Therefore, a comprehensive analysis showed that M3 had the best-fitting effect, and the leisure activities of older Chinese adults were divided into three latent classes.

**Table 1:** Optimal model fits for class selection.

	**AIC**	**BIC**	**aBIC**	**Entropy**	**LMRT(*P*)**	**BLRT(*P*)**	**Probability distributions**
M1	8,004.96	8,093.82	8,020.86	–	–	–	1
M2	7,811.18	7,992.77	7,843.67	0.874	0.001	< 0.001	0.73/0.27
M3	7,688.67	7,962.99	7,737.75	0.805	0.022	< 0.001	0.55/0.21/0.24
M4	7,633.16	8,000.21	7,698.83	0.798	0.098	< 0.001	0.19/0.15/0.19/0.47
M5	7,607.93	8,067.70	7,690.19	0.815	0.716	< 0.001	0.12/0.13/0.18/0.38/0.19

AIC, Akaike information criteria; BIC, Bayesian information criteria; aBIC, Adjusted Bayesian information criteria; LMRT, Lo-Mendell-Rubin likelihood ratio test; BLRT, Bootstrapped likelihood ratio test.

**Figure 1:**
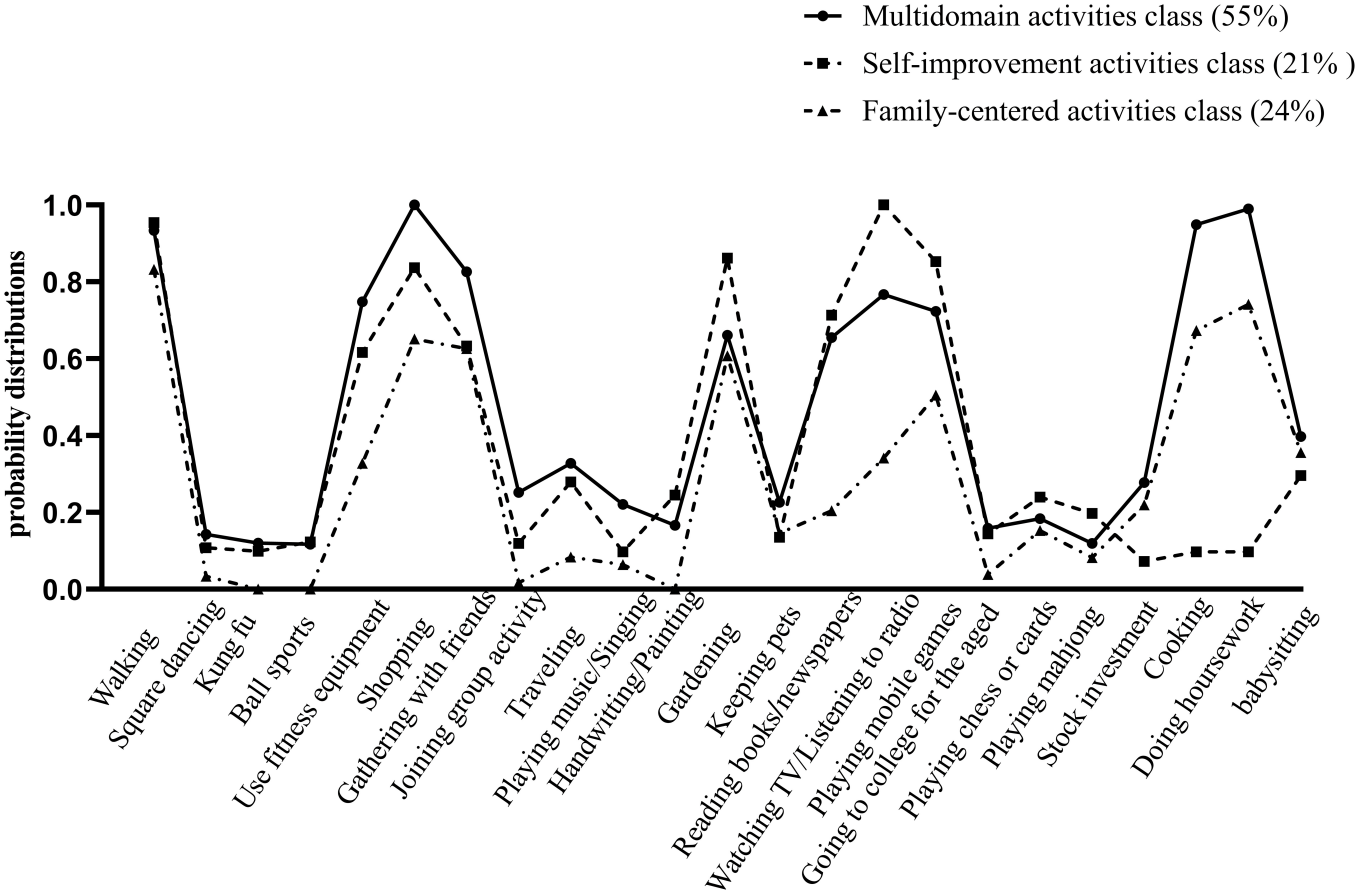
Older adults' leisure activity patterns by latent class analysis.

The conditional probability distributions were plotted based on the conditional probabilities of each class on 23 activities (Figure 1). There were 195 older adults (55%) in class 1. In class 1, older adults had a higher probability of participating in various leisure activities; thus, we named it the multidomain activities (MDA) class. There were 74 older adults (21%) in class 2, and older adults in this class participated in self-improvement activities, such as reading books/newspapers, playing mobile games/computers, and watching TV/listening to the radio. Hence, we named it the self-improvement activities (SIA) class. There are 83 older adults (24%) in class 3, and older adults in this class mostly engaged in more family-centered activities, such as cooking, housework, and gardening. Therefore, we named it the family-centered activities (FCA) class.

### 3.3. Differences among three latent classes in general characteristics

Univariate analysis showed that there were significant differences in gender, occupation, education, and BMI among the three latent classes of leisure activities (*P* < 0.001, *P* < 0.001, *P* < 0.001, *P* = 0.018; Table 2). Further multivariate logistic regression analysis showed that compared with the MDA class, older male adults were more likely to be classified as SIA class, and the possibility of male adults being classified as SIA class was 7.966 times higher than that of female adults (β = 0.275, OR = 7.966, *P* < 0.001). Compared with the MDA class, older adults in the SIA class had higher BMI (β = 0.159, OR = 1.172, *P* = 0.002). The educational level of older adults in the FCA class was significantly lower than that in the MDA class (β = −0.156, OR = 0.856, *P* < 0.001; Table 3).

**Table 2:** Differences of three latent classes in general characteristics (univariate analysis).

**Variables**	**MDA class (*n* = 195)**	**SIA class (*n* = 74)**	**FCA class (*n* = 83)**	**Statistics (*F/χ^2^/H*)**	***P***
**Gender** ^a^				36.160	< 0.001
Male	96 (49.2%)	65 (87.8%)	40 (48.2%)		
Female	99 (50.8%)	9 (12.2%)	43 (51.8%)		
Age^b^	71 (68, 74)	72 (68, 76)	70 (68, 73)	2.140	0.343
Education^b^	9 (6, 12)	9 (6.75, 12)	6 (4, 9)	27.078	< 0.001
**Occupation** ^a^				16.892	< 0.001
Mental labor	110 (56.4%)	47 (63.5%)	26 (31.3%)		
Manual labor	85 (43.6%)	27 (36.5%)	57 (68.7%)		
BMI^c^	24.98 ± 3.22	26.20 ± 3.08	25.28 ± 2.98	4.061	0.018
**Hypertension** ^a^				2.716	0.257
Yes	81 (41.5%)	38 (51.4%)	41 (49.4%)		
No	114 (58.5%)	36 (48.6%)	42 (50.6%)		
**Diabetes** ^a^				1.157	0.561
Yes	46 (23.6%)	23 (31.1%)	20 (24.1%)		
No	149 (76.4%)	51 (68.9%)	63 (75.9%)		
**MCI prevalence** ^a^				27.977	< 0.001
MCI	25 (12.8%)	9 (12.2%)	32 (38.6%)		
NC	170 (87.2%)	65 (87.8%)	51 (61.4%)		

MDA, Multidomain activities; SIA, Self-improvement activities; FCA, Family-centered activities.

^a^Presented as number (percentage),

^b^presented as median (interquartile), and

^c^presented as mean ± standard deviation. P < 0.05 was considered as significant.

**Table 3:** Differences of three latent classes in general characteristics (multivariate logistic regression analysis).

**Independent variable**	**SIA class vs. MDA class**			**FCA class vs. MDA class**		
	β	**OR (95% CI)**	***P***	β	**OR (95% CI)**	***P***
**Gender**						
Female		1			1	
Male	2.075	7.966 (3.494, 18.160)	< 0.001	0.275	1.316 (0.733, 2.363)	0.358
**Occupation**						
Mental labor		1			1	
Manual labor	−0.095	0.910 (0.449, 1.843)	0.793	0.545	1.72 (0.915, 3.248)	0.092
Education	−0.006	0.994 (0.906, 1.091)	0.898	−0.156	0.856 (0.786, 0.932)	< 0.001
BMI	0.159	1.172 (1.061, 1.296)	0.002	0.029	1.029 (0.941, 1.126)	0.527

MDA, Multidomain activities; SIA, Self-improvement activities; FCA, Family-centered activities. Model significance: P < 0.001.

### 3.4. Differences among the three latent classes in the prevalence of MCI and various cognitive domains

Gender, education, and BMI were adjusted for binary logistic regression models. First, we set dummy variables with the MDA class as a reference. The results showed that the prevalence of MCI in older adults in the FCA class was 3.694 times higher than that of the MDA class (OR = 3.694, *P* < 0.001). Afterward, we set dummy variables with the SIA class as a reference, and the results showed that the prevalence of MCI in older adults in the FCA class was 2.573 times higher than that of the SIA class (OR = 2.573, *P* = 0.036; Table 4).

**Table 4:** Binary logistic regression analysis of the association between three latent classes and the prevalence of MCI.

**Latent class**	**Model**				
	β	**SE**	**Wald** χ^2^	**OR (95% CI)**	***P***
**MDA class**				1	
SIA class	0.361	0.449	0.647	1.435 (0.595, 3.463)	0.421
FCA class	1.307	0.329	15.781	3.694 (1.939, 7.037)	< 0.001
**SIA class**				1	
MDA class	−0.361	0.449	0.647	0.697 (0.289, 1.681)	0.421
FCA class	0.945	0.451	4.386	2.573 (1.063, 6.232)	0.036

MDA, Multidomain activities; SIA, Self-improvement activities; FCA, Family-centered activities. Gender, education, and BMI were adjusted.

**Figure 2:**
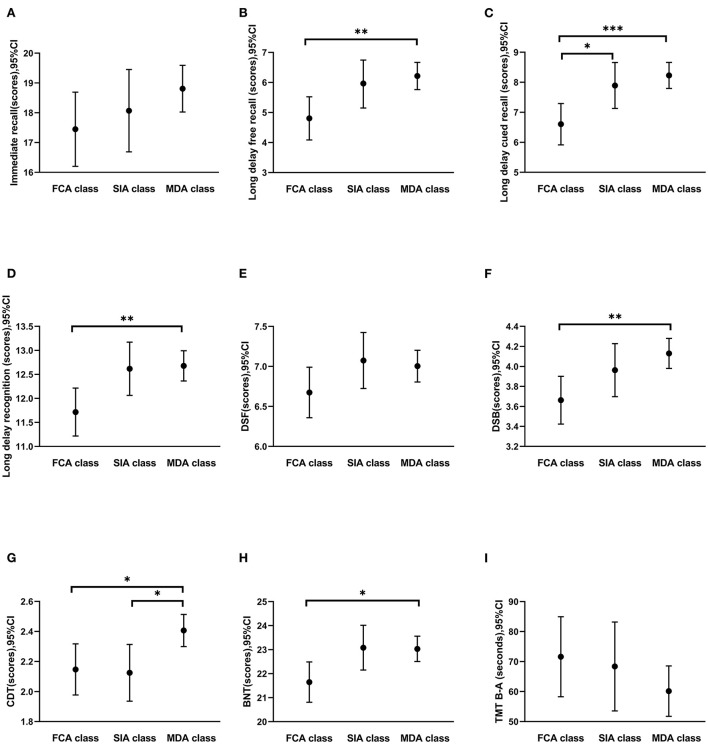
Comparison of cognitive functions among the three leisure activity patterns. **(A–D)** Memory function (immediate recall, long delay free recall, long delay cued recall, and long delay recognition); **(E, F)** attention (DSF and DSB); **(G)** visuospatial function (CDT); **(H)** language function (BNT); **(I)** executive function (TMT B-A). FCA, Family-centered activities; SIA, Self-improvement activities; MDA, Multidomain activities. Error bars represented means (95% confidence intervals). Gender, education, and BMI were controlled in the analysis of covariance. *P < 0.05, **P < 0.01, ***P < 0.001.

Figure 2 shows the comparison of all cognitive domains of older adults in different activity patterns after adjustment for gender, education, and BMI. The FCA class showed poorer performance in memory (Figures 2B–D; all *P* < 0.01), attention (Figure 2F; *P* = 0.004), language (Figure 2H; *P* = 0.037), and visuospatial function (Figure 2G; *P* = 0.021) than the MDA class. The long delay cued recall of older adults in the FCA class was lower than that in the SIA class (*P* = 0.049). The visuospatial function of older adults in the SIA class was lower than that in the MDA class (*P* = 0.037).

## 4. Discussion

Rather than focusing on one activity or one type of leisure activity, this study considered various leisure activities as a whole, divided leisure activity patterns according to the overall characteristics, and then explored the association between different leisure activity patterns and cognition. Using 23 indicators of leisure activity, the study used LCA to identify three activity patterns: MDA class, SIA class, and FCA class. Older adults in the FCA class were at high prevalence of MCI.

As expected, the participation in leisure activities of older adults was heterogeneous and assigned to three different latent classes. The largest group was the MDA class (55%). The MDA class showed a high level of participation in almost all areas of leisure activity. This pattern has also been reported in previous studies (Hong et al., 2020; Zhang et al., 2020; Katayama et al., 2021). Of older adults, 21% belonged to the SIA class. The SIA class comprised older adults who had predominantly engaged in intellectual activities. And further results showed that older males were more likely to be classified as the SIA class. This was in line with the findings of previous studies demonstrating that males were more willing to engage in some challenging and competitive intelligence activities (van Uffelen et al., 2017; Hassing, 2020). It may be because males showed significantly higher beta relative power that was responsible for active thinking than females when faced with various challenging tasks (Corsi-Cabrera et al., 1993). A total of 24% of older adults belonged to the FCA class. The FCA class showed a high level of participation in family-focused activities but lower attendance at intellectually demanding activities. This finding was consistent with a study conducted in Singapore (Hong et al., 2020). We further found that older adults with less education were more likely to be classified as FCA class. This may be because the awareness and the sense of the pleasant experience from participation in intellectual activities of older adults with low education levels were not as good as those of older adults with high education levels (Payne et al., 2011).

The results showed that among the three latent classes, older adults in the FCA class had the worst cognitive function and the highest prevalence of MCI. It was difficult to make comparisons between our current study findings and others because of the differences in the measurement of leisure activities, patterns of activity identified, and cognitive assessment tools. However, there was an apparent similarity between our study and others: older adults with high comprehensive participation had better cognitive function (Amano et al., 2018; Chen et al., 2019; Zhang et al., 2020). After controlling for the influence of possible confounding factors, from the analysis of the characteristics of activity participation types, the high prevalence of MCI in the FCA class may be because their participation in intellectual activities was significantly lower than that of the other two classes. The protective effect of intellectual activities on the cognition of the elderly was recognized by many scholars (Yates et al., 2016; Hyun et al., 2020). The cognitive reserve hypothesis posits that leisure activity can protect cognition by increasing cognitive reserve and providing cognitive resilience against brain injury (Stern, 2002). Intellectual activity can protect cognitive function in older adults by increasing the effectiveness and plasticity of neural networks (Stern, 2012; Barker et al., 2021), preventing or slowing the deposition of amyloid-β (Aβ) plaques (Landau et al., 2012). However, as a non-exercise physical activity, there is no consistent conclusion on the relationship between housework and cognition. Yee et al. (2021) have demonstrated that housework was associated with better cognitive function in older adults. Even if the amount of housework was less than recommended by exercise guidelines, the accumulation of these simple activities could increase gray matter volume and improve cognitive function in older adults (Halloway et al., 2019). However, a longitudinal study has shown that housework, such as cooking and gardening, was associated with a decline in spatial ability and memory in older adults (Hassing, 2020). A further study should explore the independent relationship between housework and cognitive function through follow-up to provide evidence in this regard.

Older adults in the FCA class were the key cognitive intervention population for the future. Increasing participation in activities that have greater socialization and cognitive reserve of older adults is the focus of future research, especially increasing participation in activities with different intellectual needs. First, the awareness of activity participation of older adults with low education levels is not enough. In this regard, the community centers should increase publicity efforts in the form of some event pictures, posters, small videos, etc. Second, older adults with low education levels have a low sense of pleasure in participating in intellectually demanding activities. The Match Hypothesis states that the flow experience will be more likely if there is a match between an individual's ability and the capacity requirements for the activity (Payne et al., 2011). As an exceptionally positive state, flow experience can motivate people to perform intellectually demanding activities at a high level and to persevere in these activities over long periods (Csikszentmihalyi et al., 2005). Hence, we can assign a weight to the cognitive component of common activities of the elderly (Karp et al., 2006) and match older adults with different educational levels to participate according to the weight of the cognitive component. For example, gardening, watching TV, participating in outdoor activities, gathering with friends, and participating in some volunteer services are activities with low intellectual needs. Reading, playing chess or cards, traveling, writing, playing mobile games, etc., are activities with high intellectual needs. In the future, activities that match different intellectual needs can be based on the education level and hobbies of older adults. Particularly, older adults with low education level can participate in some activities with lower intellectual needs or replacement activities with similar cognitive components, such as sharing housework tips, growing vegetables, tailoring and repairing, etc. Additionally, previous research has found that older adults with lower education level can obtain stronger cognitive protective effects from activities such as volunteering than those with higher education (Park et al., 2019). The community can provide older adults with low education level the opportunity to participate in volunteer activities and encourage them to actively participate, such as assisting community nucleic acid testing, publicizing garbage sorting, etc., which also has positive significance for their cognition.

This study has some limitations. First, this was a cross-sectional study, limiting the extent of inference about causality. Without longitudinal data, we could not determine whether the presence of MCI predicted participation in a less intellectual activity or whether the lack of intellectual activity led to MCI. Future longitudinal studies are needed to investigate the causal relationship between leisure activity patterns and cognition. Second, the representativeness of the sample needs to be further improved. The nature of the sample could affect the number of latent classes and characteristics. We sampled only older adults in Shijiazhuang City. Moreover, when excluding hearing loss, it was only based on the self-reports of the participants or their family members, which inevitably led to the loss of some samples. Future studies should recruit more broadly representative older adults using objective assessments. Third, leisure activity engagement was self-reported and could be influenced by the recalling bias. Additionally, because of the large number of activities, we did not measure the “total longevity” of leisure activities. We believe that the overall participation of older adults in leisure activities is in a balanced state. However, the cognitive effects of engaging in an activity for 1 year should be different from those lasting 10 years. Finally, this study failed to address other covariates related to biological factors (e.g., cytokines and Aβ). In addition to social and health covariates, future studies should include these factors.

Despite its limitations, this study has important clinical implications. First, this was one of the few studies using LCA to examine leisure activity patterns among older adults. The results showed that a single or a certain type of activity might not fully reflect a person's activity status. As such, we have shown some evidence for looking at patterns of multiple activity domains rather than a single domain. Additionally, the heterogeneity of participation in leisure activities also highlighted the importance of tailoring specific intervention strategies, especially for those at higher risk of cognitive impairment. Understanding the diversity of activity engagement may spur service providers to develop appropriate programs for different characteristics of older adults to maximize the reach of the activity program in the target group, especially for some older adults with low education levels. Community service agencies should strengthen the publicity effect and fully promote the participation of older adults in diverse activities, especially intellectual activities. Finally, it also helps practitioners and policymakers be aware of these heterogeneous behaviors, thereby maximizing the reach and meaning of leisure activities.

## Data availability statement

The raw data supporting the conclusions of this article will be made available by the authors, without undue reservation.

## Ethics statement

This study was approved by the Ethics Committee of Hebei Medical University (ethical approval number 2021080) and written informed consent was obtained from each subject before the experiment. The patients/participants provided their written informed consent to participate in this study.

## Author contributions

YZ and YL conceptualized and designed the study and the investigation. XY, LG, and BC distributed and collected questionnaires together with YZ. YZ drafted the original manuscript. YZ, XX, XM, and XY performed the initial analyses, interpreted the data, and reviewed and revised the manuscript. YL and XX obtained funding for the research and supervised the procedure of the whole investigation to evaluate the project. All authors contributed to the article and approved the submitted version.
